# Target Quantification and Semi-Target Screening of Undesirable Substances in Pear Juices Using Ultra-High-Performance Liquid Chromatography-Quadrupole Orbitrap Mass Spectrometry

**DOI:** 10.3390/foods9070841

**Published:** 2020-06-28

**Authors:** Alfonso Narváez, Yelko Rodríguez-Carrasco, Luana Izzo, Luigi Castaldo, Alberto Ritieni

**Affiliations:** 1 Department of Pharmacy, University of Naples “Federico II”, Via Domenico Montesano 49, 80131 Napoli, Italy; alfonso.narvaezsimon@unina.it (A.N.); luana.izzo@unina.it (L.I.); luigi.castaldo2@unina.it (L.C.); alberto.ritieni@unina.it (A.R.); 2 Laboratory of Food Chemistry and Toxicology, Faculty of Pharmacy, University of Valencia, Av. Vicent Andrés Estellés s/n, Burjassot, 46100 València, Spain

**Keywords:** pear juice, mycotoxins, pesticides, *Fusarium*, Q-Exactive Orbitrap

## Abstract

Fruit juices are common products in modern diets due to the supply of vegetal nutrients combined with its tastiness. Nevertheless, potential contaminants, such as mycotoxins and pesticides, can be present in commercial products due to a potential carry-over. Therefore, the aim of this study was to investigate for the first time the presence of 14 *Fusarium* mycotoxins using a quick, easy, cheap, effective, rugged, and safe (QuEChERS)-based extraction followed by an ultra-high-performance liquid chromatography-quadrupole Orbitrap high-resolution mass spectrometry in 21 pear juice samples from Italian markets. Up to nine different mycotoxins were detected, particularly an extensive presence of zearalenone (67%, *n* = 21, mean value = 0.88 ng/mL). Emerging *Fusarium* mycotoxins enniatins B, B1, A, and A1 were also detected. Additionally, 77 pesticide residues were tentatively identified through a retrospective analysis based on a mass spectral library. The prevalent presence of some non-approved pesticides, such as ethoxyquin (64%, *n* = 21) and triazophos (55%, *n* = 21), must be highlighted. The results obtained indicate an extensive contamination of marketed pear juice with undesirable compounds, and they should be taken into consideration when performing risk assessment studies.

## 1. Introduction

During the last years, diets have gravitated to higher intakes of fruits and vegetables, mainly due to its beneficial effects on health status and its protective role against chronic diseases [1]. In this line, fruit juices have become an appealing alternative, recommended as a good vitamin C source for children, and have been introduced as part of breakfast in conventional diets [2]. According to the European Fruit Juice Association (AIJN), juice consumption was 9.2 billion liters in 2017, with pear juice being one of the most consumed flavors in several countries, such as Italy [3]. The frequent intake of pear juice demands strict quality controls, especially when children become an important target group, in order to ensure safe consumption.

Several harmful compounds, originally present in pears, can also be present in marketed pear juice due to a potential carry-over during the manufacturing process. Among all the different contaminants in pears, mycotoxins and pesticide residues are some of the most impactful [4]. Mycotoxins are fungal secondary metabolites that can display several adverse effects, such as immunosuppression, carcinogenicity, or nephrotoxicity, among others [5]. In pears, the most relevant mycotoxin-producing genera are *Alternaria, Aspergillus*, and *Penicillium* [4], so analytical methods have been focused on the detection of their respective mycotoxins. Patulin (PAT), produced by *Aspergillus* and *Penicillium* spp., is the most studied mycotoxin in pear juice. Diverse effects have been attributed to PAT, including hepatotoxicity or neurotoxicity, among others [6]. Furthermore, citrinin (CIT) and ochratoxin A (OTA), produced by *Aspergillus* and *Penicillium* spp. too, and even *Alternaria* mycotoxins, such as alternariol (AOH) and alternariol monomethyl ether (AME), have been studied in this matrix [7,8]. Apart from these genera, *Fusarium* has also been classified as another pathogenic fungus in pear, so its presence was recently reported for the first time, causing postharvest decay [9].

On the other hand, pesticide residues include a broad range of toxic compounds widely used to prevent crops from pests. Nevertheless, a routine application can lead to the accumulation of residues in plants meant for human consumption, causing severe adverse effects, like neurotoxicity, carcinogenicity, and reproductive and developmental disorders [10]. In pear, these products are mainly used to avoid postharvest diseases caused by fungi, so pesticides from the benzimidazole group are commonly used. Similarly, pyrethroids represent another group of pesticides routinely applied due to its insecticidal capacity [11]. Despite the accumulation of residues being due to the intended use of pesticides, there are other factors to take into consideration, such as a potential run-off from contaminated soils and waters or even cross-contamination between different crops. Consequently, the overall pesticide profile of crops can vary from what it is expected to be. In terms of regulation, maximum residue limits (MRLs) have been established by the European Commission in pear or pear juice. Pesticide residues are brought under Regulation (EC) No. 396/2005 [12], but no specific MRLs have been set for pear juice so those corresponding to pears are applied instead. Referring to mycotoxins, Regulation (EC) No. 1881/2006 [13] covers contamination in pear juice, setting an MRL at 50 ng/mL for PAT. Several studies have reported the presence of PAT [14,15,16] and pesticide residues in pear juice [11,17,18], but no literature regarding *Fusarium* mycotoxins is available. Considering that *Fusarium* spp. has previously been identified as another pathogen in pears, its own mycotoxins could be expected in pear-derived products, even coexisting with other contaminants, like pesticides. Consequently, it is necessary to evaluate the contamination profile of pear juice even more when children represent one of the largest targets.

To overcome this, powerful analytical tools are needed. Concerning the extractive procedures, the most recent studies have used dispersive liquid-liquid microextraction (DLLME) [7], liquid-liquid extraction (LLE) [8,14], and QuEChERS (quick, easy, cheap, effective, rugged, and safe) combined with (DLLME) [11] for the extraction of contaminants from pear juice. Analytical methods include high-performance liquid chromatography-fluorescence detection (HPLC-FD) [7], HPLC-ultraviolet-visible detection (HPLC-UV-VIS) [8], HPLC-UV detection [14], and gas chromatography-electron capture detection (GC–ECD) [11]. Based on its high-resolution power, sensitivity, and accurate mass measurement, high-resolution mass spectrometry represents an optimal choice for evaluating trace contaminants occurring in complex matrices. Hence, the aim of the present study was to evaluate the presence of pesticide residues and mycotoxins produced by *Fusarium* spp. in 21 pear juice samples available in Italian markets, using ultra-high-performance liquid chromatography coupled to high-resolution Orbitrap mass spectrometry. To achieve this, an extractive methodology was validated for identifying and quantifying 14 *Fusarium* mycotoxins, followed by a screening of 283 pesticides. To the best of the authors’ knowledge, this is the first multi-class analysis including *Fusarium* toxins in marketed pear juice.

## 2. Materials and Methods

### 2.1. Chemicals and Reagents

All solvents, acetonitrile (AcN), methanol (MeOH), and water (LC-MS grade), were purchased from Merck (Darmstadt, Germany). Formic acid (MS grade) was acquired from Carlo Erba reagents (Cornaredo, Italy), whereas ammonium formate (analytical grade) was provided by Fluka (Milan, Italy). Magnesium sulfate (MgSO_4_) (anhydrous), sodium chloride (NaCl), primary-secondary amine (PSA) (analytical grade), and octadecyl carbon chain-bonded silica (C18) (analytical grade) were obtained from Sigma Aldrich (Milan, Italy). Syringe filters with polytetrafluoroethylene membrane (PTFE, 15 mm, diameter 0.2 μm) were purchased from Phenomenex (Castel Maggiore, Italy). Conical centrifuge polypropylene tubes of 50 and 15 mL were acquired from BD Falcon (Milan, Italy).

Mycotoxin standards and metabolites (purity ≥98%), namely neosolaniol (NEO), HT-2 toxin, α-zearalanol (α-ZAL), α-zearalenol (α-ZEL), T-2 toxin, β-zearalanol (β-ZAL), β-zearalenol (β-ZEL), zearalanone (ZAN), zearalenone (ZEN), enniatins (ENNA, ENNA1, ENNB, and ENNB1), and beauvericin (BEA), were provided by Sigma Aldrich (Milan, Italy). For the preparation of individual stock solutions, 1 mg of each mycotoxin was diluted in 1 mL of methanol. A working standard solution including all the analytes was built by diluting in MeOH:H_2_O (70:30 v/v, 0.1% formic acid) until the concentrations needed for the spiking experiments were reached: 100, 20, and 10 ng/mL. The analytical standards were kept in a tightly closed container under cool dry conditions at −20 °C in a well-ventilated place as stated in the safety data sheets provided by the manufacturer.

### 2.2. Sampling

A total of 21 pear juices samples from different European brands were randomly purchased between January and February 2020 from different supermarkets located in Campania region, southern Italy. The sampling was limited to one product per brand. The samples were divided into organic (*n* = 7) and conventional (*n* = 14) samples as indicated on the label by the manufacturer. In all cases, the percentage of fruit in the analyzed samples was above 50%; other ingredients declared in the labels from the samples were water, sugar, glucose-fructose syrup, lemon juice, and citric and ascorbic acid. All samples were stored in a refrigerator at 4°C into their original packages and analyzed within 3 days after sample registration.

### 2.3. Sample Preparation

In this work, the sample preparation procedure reported by Desmarchelier et al. [19] was selected as the starting point and slightly modified. Briefly, 10 mL of sample were placed into a 50-mL Falcon tube and mixed with 5 mL of water prior to be shaken for 1 min in a vortex. Then, 10 mL of AcN were added and the mixture was horizontally shaken for 30 min at 294× *g*. After that, 4 g of magnesium sulfate and 1 g of sodium chloride were added. The mixture was shaken by hand for 1 min and centrifuged at 4907× *g* for 10 min at 15 °C in an SL 16R centrifuge (Thermo Fisher Scientific LED GmbH, Langenselbold, Germany). Then, 3 mL of the upper acetonitrile layer were placed into a 15-mL Falcon tube containing 900 mg of magnesium sulfate, 150 mg of C18 sorbent, and 150 mg of PSA, and vortexed for 1 min. The mixture was centrifuged for 10 min at 4907× *g* at 15 °C, and 0.5 mL of the upper layer was added. Finally, the extract was evaporated to dryness under gentle nitrogen flow, reconstituted with 0.5 mL of MeOH/H_2_O (70:30 v/v; 0.1% formic acid), and filtered through a 0.2-μm filter prior to UHPLC-Q-Orbitrap HRMS analysis.

### 2.4. UHPLC-Q-Orbitrap HRMS Analysis

Chromatographic analysis was performed using an ultra-high pressure liquid chromatograph (UHPLC, Thermo Fisher Scientific, Waltham, MA, USA) equipped with a Dionex Ultimate 3000 (Waltham, MA, USA), a degassing system, an auto sampler device, a Quaternary UHPLC pump working at 1250 bar, and a thermostated (30 °C) Luna Omega (50 × 2.1 mm, 1.6 μm, Phenomenex) column. The mobile phases were water (phase A) and methanol (phase B), with both phases containing 0.1% formic acid and 5 mM ammonium formate. The separation gradient for the UHPLC-Orbitrap HRMS analyses consisted of an initial 0% of phase B kept for 1 min, and then increased up to 95% B in 1 min, followed by a hold-time of 0.5 min. Then, the gradient switched back to 75% B in 2.5 min and decreased again up to 60% B for 1 min. Finally, the gradient returned in 0.5 min at 0% B and then the column was re-equilibrated during 1.5 min at 0% B, giving a total run time of 8 min. The flow rate was 0.4 mL/min and a total of 5 µL of the samples were injected.

The UHPLC system was coupled to a Q-Exactive Orbitrap mass spectrometer. The spectrometer worked in positive and negative mode through fast polarity switching, setting two scan events (full ion MS and All ion fragmentation, AIF). Full scan data were obtained at a resolving power of 35,000 full width at half maximum (FWHM) at 200 *m/z*. The ionization parameters were capillary temperature, 290 °C; spray voltage, 4 kV (−4 kV in ESI− mode); sheath gas pressure (N_2_ > 95%), 35; S-lens radio frequency (RF) level, 50; auxiliary gas heater temperature, 305 °C; and auxiliary gas (N_2_ > 95%), 10. The selected value for the automatic gain control (AGC) target was 1e6, the injection time was set to 200 ms, and a scan range of *m/z* 100 to 1000 was selected. The scan rate was set at 2 scans/s. The parameters for the scan event of AIF were: maximum injection time, 200 ms; mass resolving power, 17,500 FWHM; ACG target, 1e5; scan time, 0.10 s; *m/z* scan range, 100–1000; retention time window, 30 s; and *m/z* isolation window, 5.0. The Orbitrap-MS parameters were optimized in a previously published article [20]. Table 1 shows the analytical parameters of the studied mycotoxins, including the elemental composition, retention time (RT), adduct ion, theoretical and measured mass, accurate mass error, collision energy, and product ion as well as tolerable daily intake (TDI). The energy was chosen when at least the parent ion was held at 10% and the generating product ions at a 90% intensity. A mass tolerance below 5 ppm was set for identification of the ions. Retrospective semi-target screening was performed using spectral data provided by a pesticide spectral library (Pesticide Spectral Library Version 1.1 for LibraryView™ Software, AB SCIEX, Framingham, MA, USA). The identification and confirmation were carried out according to the accurate mass measurement with a mass tolerance below 5 ppm for the molecular ion and for both fragments at the intensity threshold of 1000. Precursor and product ions were required to identify both targeted and nontargeted compounds. Data analysis and processing were conducted using Quan/Qual Browser Xcalibur software, version 3.1.66 (Thermo Fisher Scientific, Waltham, MA, USA).

### 2.5. Validation Parameters

In-house validation was carried out according to the guidelines established by the EU Commission Decision 2002/657/EC [21] and the SANTE criteria (SANTE/12682/2019) [22]. The method validation was based on the following parameters: Selectivity, trueness, repeatability (intra-day precision), reproducibility (inter-day precision), linearity, limit of detection (LOD), and limit of quantification (LOQ). The selectivity of the method was evaluated by analyzing blank samples (*n* = 10) to detect signals that could interfere with the analytes. The peaks for the analytes of interest in the samples were confirmed by comparing the retention times of the peak with those of standard solutions and also identifying both the precursor and product ions, with a mass tolerance below 5 ppm. To determine the linearity (*R*^2^), standard solutions built in neat solvent and matrix-matched calibration were compared by spiking blank samples with selected mycotoxins at eight concentration levels over a range of 0.4–100 ng/mL. Calibration curves were prepared in triplicate. In order to reveal the presence of matrix effects, the slopes of each linear calibration function were evaluated. The signal suppression/enhancement (%SSE) due to matrix effects was determined according to the following equation:

%SSE = S_m_/S_s_ × 100, (1)

where S_m_ is the matrix-matched calibration slope and S_s_ is the solvent calibration slope. An SSE of 100% indicates that no matrix effect occurred in the concentration range evaluated. An SSE value higher than 100% revealed signal enhancement, whereas there was signal suppression if the SSE value was below 100%. Trueness was determined by spiking three blank samples at three different levels (100, 20, and 10 ng/mL) during three non-consecutive days and the results were expressed as percentage of recovery. Values in the range 70–120% in relation to the theoretical concentrations were considered as satisfactory Intraday precision (repeatability) was expressed in terms of the relative standard deviation (RSD_r_) after comparing the recoveries from three determinations in a single day (*n* = 3) for each fortification level. Inter-day precision (reproducibility) was expressed as the relative standard deviation (RSD_R_) of a triplicated determination on three non-consecutive days (*n* = 9) for each fortification level. LODs were set considering the lowest concentration where the molecular ion could be identified (mass error value below 5 ppm) and LOQs were defined as the minimum concentration inside the linear range (mass error value below 5 ppm) with deviation below 20%.

### 2.6. Quality Assurance/Quality Control

For a proper confirmation of the peaks, the retention times corresponding to each analyte in the samples were compared to those in standard solutions at a tolerance of ± 2.5%. A mass error of 5 ppm was set for identification of both the precursor and product ions. Referring to the quality assurance/quality control (QA/QC) procedure, a reagent blank, a sample blank, and a replicate sample were put at the beginning and end of each sample batch in order to evaluate the efficacy and stability of the system throughout the whole batch. A potential carry-over was also evaluated through blank samples (*n* = 10) injected right after the highest calibration value, with 100, 20, and 10 ng/mL being the concentrations chosen for the analytical quality control.

### 2.7. Exposure Assessment

The exposure assessment was performed following a deterministic approach. Data reported by the Italian National Food Consumption Survey INRAN-SCAI 2005-06 was considered so five different age groups were made: Infants (0.1–2.9 years), mean consumption of juice of 150 mL/day; children (3–9.9 years), mean consumption of 127 mL/day; teenagers (10–17.9 years), mean consumption of 122 mL/day; adults (18–65 years), mean consumption 58 mL/day; and elderly (>65 years), mean consumption 50 mL/day. The body weight assigned to each group was 11.3, 26.1, 52.6, 69.7, and 70.1 kg, respectively. For the calculation of the probable daily intake (PDI) corresponding to each mycotoxin, the consumption data provided by the Survey INRAN-SCAI 2005–06 was combined with the contamination data here obtained, following the next equation:

PDI_m_ = (C_m_ × I)/bw, (2)

where *PDI_m_* is the probable daily intake (ng/kg bw/d) corresponding to each mycotoxin, *m*; *C_m_* is the average content of a certain mycotoxin in pear juice (ng/mL); *I* represents the intake of juice (mL); and *bw* is the body weight attached to each age group (kg). After *PDI_m_* was calculated, the risk characterization, considered as the percentage of relevant *TDI_m_*, was evaluated by dividing the resultant *PDI_m_* by its *TDI_m_* value (Table 1). Since ENNB, ENNB1, ENNA, ENNA1, and BEA do not have an assigned TDI, a theoretical 20 ng/kg bw value was used, corresponding to the lowest one for a *Fusarium* toxin:

%TDI_m_ = PDI_m_/TDI_m_ × 100. (3)

### 2.8. Statistical Analysis

The Mann–Whitney U test was used to evaluate differences between juice typology considering *p* values < 0.05 as significant. Statistical analysis of the results was carried out using IBM SPSS version 25 statistical software package (SPSS, Chicago, IL, USA).

## 3. Results

### 3.1. Analytical Method Validation

The method was validated in order to extract and quantify 14 different *Fusarium* mycotoxins in pear juice. The results are shown in Table 2 All the analytes showed good linearity, with the regression coefficients (R^2^) above 0.990 in the range evaluated (0.4–100 ng/mL), and a deviation ≤20% for each level of the calibration curves. Signal suppression/enhancement (%SSE) as a consequence of matrix interference was evaluated by comparing the curves built in neat solvent and blank matrix, with a minimal deviation (≤17%) being observed. Therefore, the external calibration curves were considered for quantification purposes. Sensitivity was assessed through the limits of quantifications (LOQs), that ranged from 0.4 to 3.1 ng/mL. To evaluate trueness, recovery studies were carried out in triplicate at three different spiking levels. Values corresponding to fortification at 100 ng/mL ranged between 70% and 106%, from 72–106% at 20 ng/mL, and from 70–103% at 10 ng/mL, meaning an efficient extraction procedure even at low concentrations levels. Precision was evaluated through both RSD_r_ and RSD_R_, showing values below 19% for all the mycotoxins analyzed. These results fulfill the criteria set by the European EU Commission Decision 2002/657/EC [21] established for a reliable quantification. This methodology, based on a simple QuEChERS extraction, stands as a powerful tool for detecting *Fusarium* mycotoxins in pear juice at low levels, reaching a higher sensitivity for several of the analytes than previous methods developed for detecting a single analyte in pear juice and carried out by Spadaro, Garibaldi, and Gullino [16] (PAT, LOQ = 1.7 ng/mL); Bonerba, Ceci, Conte, and Tantillo [15] (PAT, LOQ = 1 ng/mL); and Pan et al. [23] (AOH = 1.3 ng/mL).

### 3.2. Analysis of Real Samples

Up to nine different *Fusarium* mycotoxins were detected in the analyzed pear juice samples. In total, 20 out of 21 samples showed contamination with at least one mycotoxin, generally at low levels or even below the LOQ, as shown in Table 3. ZEN was the most frequently detected compound, present in 67% of the samples, with concentrations ranging from below the LOQ to 1.5 ng/mL. T2 was also a relevant mycotoxin in the analyzed samples, showing an incidence of 33% at concentrations from <LOQ up to 2.0 ng/mL. Similarly, HT-2 was detected in 33% of the samples, ranging from below <LOQ to 7.0 ng/mL. The main enniatins were also detected: ENNB and ENNA1 were present in 19% of the samples, ranging from <LOQ to 0.8 ng/mL and 1.2 ng/mL, respectively; ENNB1 was found in 14% of the samples at concentrations going from <LOQ up to 0.5 ng/mL; and ENNA was only found in one sample (5%) at 1.0 ng/mL. Lastly, ZEN metabolites were also observed. ZAN was present in 10% of the samples at concentrations below the LOQ, whereas α-ZAL was quantified in 14% of the samples, ranging from <LOQ to 10.5 ng/mL. To date, several studies have only evaluated the presence of PAT in Italian pear juice as a consequence of *Penicillium expsansum* contamination, which is the main fungus causing postharvest diseases. Spadaro, Garibaldi, and Gullino [16] reported an incidence of 64% (*n* = 39), with 17 samples showing a contamination below 10 ng/mL and 8 samples above 10 ng/mL. Similarly, Bonerba, Ceci, Conte, and Tantillo [15] found patulin in 40% of the pear juice samples (*n* = 35) at concentrations ranging from 5 to 92 ng/mL. Recently, *Alternaria* mycotoxins have been studied in pear and pear-derived foodstuffs. Pan, Sun, Pu, and Wei [23] investigated *Alternaria* toxin AOH in fresh pears (*n* = 5), observing an absence of contamination despite having good sensitivity (LOQ = 1.3 ng/mL). A specific methodology for detecting AOH and AME in pear juice has been developed by Ruan, Diao, Zhang, Zhang, and Liu [7]. The results obtained here show that the *Fusarium* toxin ZEN extensively occurred in pear juice samples, having a larger incidence than the one reported for PAT or AOH in the mentioned studies, whereas other less detected mycotoxins, such as T2 or HT-2, also had a considerable impact.

Bearing in mind the type of sample, significant differences (*p* < 0.05) were found when comparing the occurrence of mycotoxins in both organic and conventional juice, being more frequent in organic samples, as expected. Additionally, co-occurrence of mycotoxins was observed in high frequency in organic juice samples. Up to 65% of the conventional juice samples (*n* = 14) showed contamination with only one mycotoxin, whereas four or more mycotoxins co-occurred in the majority of organic samples (71%, *n* = 7). The most common associations were ZEN alongside its two metabolites, and ZEN, T-2, and ENNB, which seems to be a frequent mixture in several plant-based foodstuffs [24,25]. Furthermore, ENNB1 and ENNA1 co-occurred with ENNB in organic samples, and the combination ENNA and ENNA1 was also observed in one conventional juice sample. The presence of multiple mycotoxins could affect its toxicological potential, deriving into synergic or additive effects as observed in in vitro assays [26]. Based on the above-discussed points and considering the popular trend of organic and environment-friendly products, *Fusarium* mycotoxins should also be taken into consideration in exposure assessment studies involving pear and pear juices. Moreover, further toxicological knowledge in terms of the combination of food contaminants is needed in order to ensure safe consumption.

### 3.3. Exposure Assessment

As reflected by the Italian National Food Consumption Survey INRAN-SCAI 2005-06, juices are mostly consumed by the young population, so a bigger intake accounts for a higher risk. Considering that *Fusarium* mycotoxins have not been studied in pear juice, the exposure to these mycotoxins might be underestimated, so an exposure assessment and risk characterization were performed.

Table 4 summarizes the risk characterization of the mycotoxins found in the juice samples. The mean content of mycotoxins was 2.88, 0.88, and 0.25 ng/mL for ZEN + α-ZAL, T-2 + HT-2, and enniatins, respectively. Among the different age groups, the probable daily intake strongly varied. Infants were identified as the group with the highest PDIs due to a heavier consumption of juice and a lower body weight. Values corresponding to the rest of the groups ranged from 2 to 20 times lower in comparison with the infants’ results. These PDIs values are below the TDIs established by the Scientific Committee on Food of the European Commission, set as 250 ng/kg bw/day for the sum of ZEN and its derived products, 20 ng/kg bw/day for the sum of T-2 HT-2, and a theoretical value of 20 ng/kg bw/day for the sum of enniatins. Considering the results, the pear juices analyzed here account for 0.78% to 14.65% of the TDI for ZEN + α-ZAL, from 3% to 55.95% of the TDI set for T-2 and HT-2, and from 0.85% to 15.90% for enniatins. This suggests that the exposure to *Fusarium* mycotoxins as a consequence of juice consumption might not represent a health concern, but the intake of mycotoxins by infants due to regular consumption could be of importance. Therefore, the results suggest having a watchful attitude in order to ensure safe consumption.

### 3.4. Identification of Non-Target Compounds through Retrospective Analysis in Studied Samples

**Figure 1:**
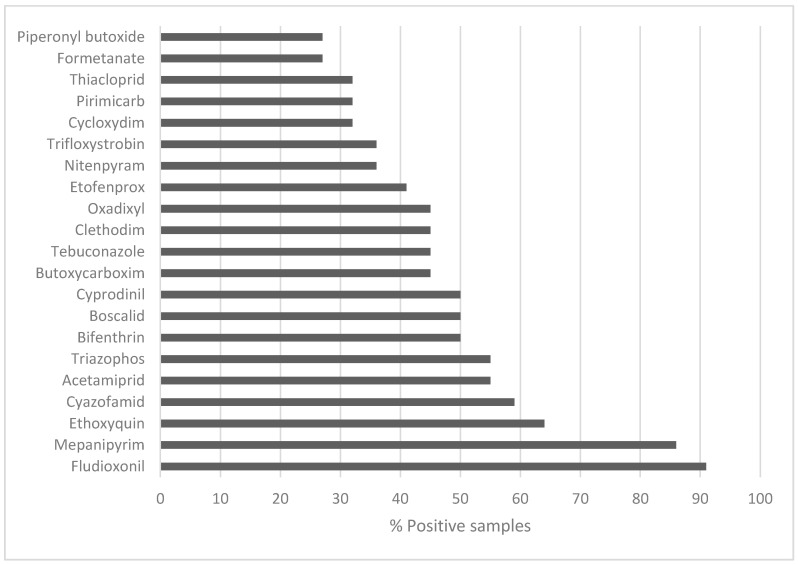
Occurrence of non-target pesticides in pear juice samples after retrospective screening.

The semi-target screening was performed using a mass spectral library, allowing the detection of 283 different pesticide residues in the analyzed samples. The pesticides present in more than 25% of the samples are shown in Figure 1. Up to 77 pesticide residues were tentatively identified, but the presence of several compounds that have not yet been approved by the EU should be noted. Ethoxyquin was detected in 64% of the samples (*n* = 21), being the third most frequently found residue. This pesticide acts as a fungicide during the postharvest stage of the crops through its scald-preventive properties [27]. Currently, the use of ethoxyquin is suspended by European Commission Decision 2011/143/EU [28]. Triazophos is an insecticide, which was found in 55% of the samples (*n* = 21). The use of products containing this compound is not allowed under Regulation No. 1107/2009 [29] due to its toxicity, as reported by the European Food Safety Authority (EFSA) [30]. Similarly, the insecticide bifenthrin was also detected in 50% of the samples (*n* = 21). Although bifenthrin would fulfil the safety requirements according to the last update of report SANCO/12946/2011 released in 2018, it has not received any authorization yet. Oxadixyl was another relevant compound in the analyzed samples. This fungicide was found in 46% of the samples (*n* = 21) despite its use not allowed being in pears, as brought under Regulation (EC) No. 2076/2002 [31]. According to the mentioned legislation, butoxycarboxim was established as another forbidden insecticide, but it was present in 46% of the samples (*n* = 21).

Considering that pesticide residues occurred in all samples, organic juice samples showed significantly less (*p <* 0.05) residues than conventional samples, as expected. This fact could also explain the extensive contamination of organic samples in comparison with conventional samples. Therefore, the presence of non-approved pesticides in the analyzed samples indicates the necessity of monitoring potential contaminants of pears and pear-derived products.

## 4. Conclusions

A QuEChERS-based extraction was validated in order to detect and quantify 14 *Fusarium* mycotoxins for the first time in pear juice, using ultra-high-performance liquid chromatography coupled to high-resolution Q-Orbitrap mass spectrometry. The extraction fulfilled all the criteria set by the EU in terms of linearity, trueness, specificity, selectivity, and precision. This methodology was then applied to 21 pear juice samples purchased from Italian markets. Up to 95% of the samples showed mycotoxin contamination, indicating the extensive presence of ZEN and its metabolites and T-2 and its metabolites. The emerging *Fusarium* mycotoxins enniatins B, B1, A, and A1 were also detected in the samples. Organic juice samples showed a significantly higher contamination (*p <* 0.05), with at least four mycotoxins co-occurring in 71% of the samples (*n* = 7), when compared to conventional ones, with only one mycotoxin per sample in 65% of the cases (*n* = 14). Additionally, 77 pesticide residues were tentatively identified through a retrospective analysis based on semi-target screening. The prevalent presence of some non-approved pesticides, such as ethoxyquin (64%, *n* = 21) and triazophos (55%, *n* = 21), must be highlighted. The results obtained highlight an extensive contamination of marketed pear juice with undesirable compounds, including mycotoxins and pesticide residues. Hence, cumulative risk characterization studies of undesirable substances with chronic effects need to be performed, as well as more realistic risk assessment studies. Considering that children represent one of the largest targets for juices alongside the uprising trend of environmental-friendly products, there is a necessity of evaluating the contamination profile of these products to ensure safe consumption.

## Figures and Tables

**Table 1:** Ultra-high-performance liquid chromatography-high-resolution mass spectrometry (UHPLC-HRMS) parameters and tolerable daily intakes (TDIs) corresponding to the evaluated mycotoxins.

Analyte	Retention time (min)	Elemental Composition	Adduction	Theoretical Mass (*m/z)*	Measured Mass (*m/z*)	Accuracy (Δppm)	Collision Energy (eV)	Product Ions (*m/z*)	TDI (ng/kg bw)
NEO	4.25	C_19_H_26_O_8_	(M + NH_4_)^+^	400.19659	400.19632	−0.67	10	305.13803 141.00530	n.d. ^a^
HT-2	4.74	C_22_H_32_O_8_	(M + NH_4_)^+^	442.24354	442.24323	−0.7	27	263.12744 215.10641	20 ^b^
α-ZAL	4.81	C_18_H_26_O_5_	(M − H)^−^	321.17044	321.17065	0.65	29	259.09497 91.00272	250 ^c^
α-ZEL	4.83	C_18_H_24_O_5_	(M − H)^−^	319.15510	319.15500	−0.31	36	174.95604 129.01947	250 ^c^
T-2	4.85	C_24_H_34_O_9_	(M + NH_4_)^+^	484.25411	484.25430	0.39	23	215.10603 185.09561	20 ^b^
β-ZAL	4.94	C_18_H_26_O_5_	(M − H)^−^	321.17044	321.17059	0.47	40	259.09497 91.00272	250 ^c^
β-ZEL	4.97	C_18_H_24_O_5_	(M − H)^−^	319.15510	319.15500	−0.31	36	174.95604 160.97665	250 ^c^
ZAN	4.98	C_18_H_24_O_5_	(M − H)^−^	319.15510	319.15491	−0.6	35	273.01187 131.05020	250 ^c^
ZEN	5.01	C_18_H_22_O_5_	(M + H)^+^	317.13945	317.13928	−0.54	−32	175.03989 131.05008	250 ^c^
ENN B	5.56	C_33_H_57_N_3_O_9_	(M + NH_4_)^+^	657.44331	657.44348	0.26	50	214.14320 196.13280	n.d. ^a^
ENN B1	5.68	C_34_H_59_N_3_O_9_	(M + NH_4_)^+^	671.45986	671.45935	−0.76	48	214.14343 196.13295	n.d. ^a^
BEA	5.73	C_45_H_57_N_3_O_9_	(M + NH_4_)^+^	801.44331	801.44318	−0.16	70	262.76715 244.18239	n.d. ^a^
ENN A1	5.82	C_35_H_61_N_3_O_9_	(M + NH_4_)^+^	685.47461	685.47449	−0.18	48	228.15900 210.14847	n.d. ^a^
ENN A	5.99	C_36_H_63_N_3_O_9_	(M + NH_4_)^+^	699.49026	699.48987	−0.56	43	228.15900 210.14847	n.d. ^a^

^a^ not determined; ^b^ sum of T-2 and HT-2; ^c^ sum of ZEN and its forms α-ZEL, β-ZEL, α-ZAL, β-ZAL, and ZAN in terms of ZEN equivalents being 60, 0.2, 4, 2, and 1.5, respectively, its molar potency factors. Neosolaniol (NEO), HT-2 toxin, α-zearalanol (α-ZAL), α-zearalenol (α-ZEL), T-2 toxin, β-zearalanol (β-ZAL), β-zearalenol (β-ZEL), zearalanone (ZAN), zearalenone (ZEN), enniatins (ENNA, ENNA1, ENNB, and ENNB1), and beauvericin (BEA).

**Table 2:** Method performance: linearity, matrix effect (SSE %), recovery, and limit of quantification (LOQ).

Analyte	Linearity (*R*^2^)	SSE (%)	Recovery (%)			Precision (%) (RSD_r_, (RSD_R_)			LOQ (ng/mL)
			100 ng/mL	20 ng/mL	10 ng/mL	100 ng/mL	20 ng/mL	10 ng/mL	
NEO	0.9971	98	99	99	91	5 (13)	8 (18)	16 (12)	1.6
HT-2	0.9967	104	88	89	72	13 (13)	15 (13)	14 (17)	1.6
A-ZAL	0.9944	83	84	77	72	10 (8)	10 (8)	7 (7)	3.1
A-ZOL	0.9967	90	97	93	102	15 (19)	8 (18)	10 (19)	3.1
T-2	0.9998	105	106	106	103	11 (14)	16 (18)	7 (17)	1.6
B-ZAL	0.9941	113	93	85	89	9 (12)	13 (16)	12 (16)	1.6
B-ZOL	0.9997	112	81	87	77	10 (8)	8 (7)	8 (6)	0.8
ZAN	0.9993	118	83	85	77	7 (5)	13 (9)	6 (5)	0.4
ZON	0.9994	117	84	91	87	5 (5)	4 (4)	6 (8)	0.4
ENN B	0.9995	103	73	76	71	5 (8)	7 (5)	10 (7)	0.8
ENN B1	0.9980	94	76	81	75	10 (9)	8 (9)	8 (8)	0.4
BEA	0.9977	96	78	84	78	5 (5)	10 (6)	15 (10)	1.6
ENN A1	0.9994	101	70	74	71	3 (6)	7 (5)	13 (9)	0.8
ENN A	0.9994	103	70	72	70	3 (2)	7 (5)	6 (4)	0.8

RSD_r_: repeatability relative standard deviation; RSD_R_: reproducibility relative standard deviation

**Table 3:** Incidence and range of concentrations of the mycotoxins detected in conventional and organic pear juice samples.

Juice Typology (n)	ZEN		ZAN		A-ZAL		T-2		HT-2		ENNB		ENNB1		ENNA		ENNA1	
	Incidence (n (%))	Range (ng/mL)	Incidence (n (%))	Range (ng/mL)	Incidence (n (%))	Range (ng/mL)	Incidence (n (%))	Range (ng/mL)	Incidence (n (%))	Range (ng/mL)	Incidence (n (%))	Range (ng/mL)	Incidence (n (%))	Range (ng/mL)	Incidence (n (%))	Range (ng/mL)	Incidence (n (%))	Range (ng/mL)
Conventional juice (14)	7 (50)	<L ^2^ - 1.5	0 (0)	nd ^1^	1 (7)	10.5	2 (14)	<L	4 (29)	<L - 7.0	0 (0)	nd	1 (7)	<L	1 (7)	1.0	3 (21)	<L - 1.2
Organic juice (7)	7 (100)	<L - 0.6	2 (29)	<L	2 (29)	<L - 3.5	5 (71)	<L - 2.0	3 (43)	<L - 1.6	4 (57)	<L - 0.8	2 (29)	<L - 0.5	0 (0)	nd	1 (14)	0.8
Total	14 (67)	<L - 1.5	2 (10)	<L	3 (14)	<L - 10.5	7 (33)	<L - 2.0	7 (33)	<L - 7.0	4 (19)	<L - 0.8	3 (14)	<L - 0.5	1 (5)	1.0	4 (19)	<L - 1.2

^1^ Not determined; ^2^ Limit of quantification.

**Table 4:** Risk characterization of mycotoxins found in pear juice samples according to the tolerable daily intake values.

Mycotoxins	C_m_ (ng/mL)	Probable Daily Intake (PDI) (ng/kg bw/d)					Risk Characterization (%TDI)				
		Infants	Children	Teenager	Adult	Elderly	Infants	Children	Teenager	Adult	Elderly
ZEN + α-ZAL	2.88	38.24	14.01	6.69	2.3	2.04	15.3	5.6	2.68	0.92	0.82
T-2 + HT-2	0.88	11.68	4.28	2.04	0.7	0.62	58.40	21.40	10.20	3.50	3.10
ENNB + ENNB1 + ENNA + ENNA1	0.25	3.32	1.22	0.58	0.2	0.18	16.60	6.10	2.90	1.00	0.90

## References

[1] Woodside J.V., Young I.S., McKinley M.C. (2013). Fruits and vegetables: Measuring intake and encouraging increased consumption. Proc. Nut. Soc..

[2] Heyman M.B., Abrams S.A. (2017). Fruit Juice in Infants, Children, and Adolescents: Current Recommendations. Pediatrics.

[3] European Fruit Juice Association Liquid Fruit Market Report - 2017. Belgium, Brussels. https://aijn.eu/files/attachments/.598/2018_Liquid_Fruit_Market_Report.pdf.

[4] Mandappa I.M., Basavaraj K., Manonmani H.K., Rajauria G., Tiwari B.K. (2018). Analysis of Mycotoxins in Fruit Juices. Fruit Juices.

[5] Marin S., Ramos A.J., Cano-Sancho G., Sanchis V. (2013). Mycotoxins: Occurrence, toxicology, and exposure assessment. Food Chem. Toxicol..

[6] Pal S., Singh N., Ansari K.M. (2017). Toxicological effects of patulin mycotoxin on the mammalian system: An overview. Toxicol. Res..

[7] Ruan C., Diao X., Zhang H., Zhang L., Liu C. (2016). Development of a dispersive liquid–liquid microextraction technique for the analysis of citrinin, alternariol and alternariol monomethyl ether in fruit juices. Anal. Methods.

[8] Oteiza J.M., Khaneghah A.M., Campagnollo F.B., Granato D., Mahmoudi M.R., Sant’Ana A.S., Gianuzzi L. (2017). Influence of production on the presence of patulin and ochratoxin A in fruit juices and wines of Argentina. LWT.

[9] Wenneker M., Pham K.T.K., Lemmers M.E.C., de Boer F.A., van der Lans A.M., van Leeuwen P.J., Hollinger T.C., Thomma B.P.H.J. (2016). First Report of Fusarium avenaceum Causing Postharvest Decay on ‘Conference’ Pears in the Netherlands. Plant Dis..

[10] Mostafalou S., Abdollahi M. (2017). Pesticides: An update of human exposure and toxicity. Arch. Toxicol..

[11] Zhang Y., Zhang X., Jiao B. (2014). Determination of ten pyrethroids in various fruit juices: Comparison of dispersive liquid–liquid microextraction sample preparation and QuEChERS method combined with dispersive liquid–liquid microextraction. Food Chem..

[12] European Commission (2005). Regulation (EC) No. 396/2005 of the European Parliament and of the Council of 23 February 2005 on maximum residue levels of pesticides in or on food and feed of plant and animal origin and amending Council Directive 91/414/EEC. Off. J. Eur. Union.

[13] European Commission (2006). Commission Regulation (EC) No. 1881/2006 of the European Parliament and the Council of 19 December 2006 setting maximum levels for certain contaminants in foodstuffs. Off. J. Eur. Union.

[14] Zouaoui N., Sbaii N., Bacha H., Abid-Essefi S. (2015). Occurrence of patulin in various fruit juice marketed in Tunisia. Food Control.

[15] Bonerba E., Ceci E., Conte R., Tantillo G. (2010). Survey of the presence of patulin in fruit juices. Food Addit. Contam. B.

[16] Spadaro D., Garibaldi A., Gullino M.L. (2008). Occurrence of patulin and its dietary intake through pear, peach, and apricot juices in Italy. Food Addit. Contam. B.

[17] Ferrer C., Martínez-Bueno M.J., Lozano A., Fernández-Alba A.R. (2011). Pesticide residue analysis of fruit juices by LC–MS/MS direct injection. One year pilot survey. Talanta.

[18] Du J., Yan H., She D., Liu B., Yang G. (2010). Simultaneous determination of cypermethrin and permethrin in pear juice by ultrasound-assisted dispersive liquid-liquid microextraction combined with gas chromatography. Talanta.

[19] Desmarchelier A., Mujahid C., Racault L., Perring L., Lancova K. (2011). Analysis of Patulin in Pear- and Apple-Based Foodstuffs by Liquid Chromatography Electrospray Ionization Tandem Mass Spectrometry. J. Agr. Food Chem..

[20] Castaldo L., Graziani G., Gaspari A., Izzo L., Tolosa J., Rodríguez-Carrasco Y., Ritieni A. (2019). Target Analysis and Retrospective Screening of Multiple Mycotoxins in Pet Food Using UHPLC-Q-Orbitrap HRMS. Toxins.

[21] European Commission (2002). Commission Decision 2002/657/EC of 12 August 2002 implementing Council Directive 96/23/EC concerning the performance of analytical methods and the interpretation of results. Off. J. Eur Union.

[22] European Commission (2020). Analytical Quality Control and Method Validation Procedures for Pesticide Residues Analysis in Food and Feed (SANTE/12682/2019). https://www.eurl-pesticides.eu/docs/public/tmplt_article.asp?CntID=727.

[23] Pan T.-T., Sun D.-W., Pu H., Wei Q. (2018). Simple Approach for the Rapid Detection of Alternariol in Pear Fruit by Surface-Enhanced Raman Scattering with Pyridine-Modified Silver Nanoparticles. J. Agr. Food Chem..

[24] Narváez A., Rodríguez-Carrasco Y., Castaldo L., Izzo L., Ritieni A. (2020). Ultra-High-Performance Liquid Chromatography Coupled with Quadrupole Orbitrap High-Resolution Mass Spectrometry for Multi-Residue Analysis of Mycotoxins and Pesticides in Botanical Nutraceuticals. Toxins.

[25] Veprikova Z., Zachariasova M., Dzuman Z., Zachariasova A., Fenclova M., Slavikova P., Vaclavikova M., Mastovska K., Hengst D., Hajslova J. (2015). Mycotoxins in Plant-Based Dietary Supplements: Hidden Health Risk for Consumers. J. Agr. Food Chem..

[26] Smith M.-C., Madec S., Coton E., Hymery N. (2016). Natural Co-Occurrence of Mycotoxins in Foods and Feeds and Their in vitro Combined Toxicological Effects. Toxins.

[27] Pesticides Properties DataBase (PPDB) University of Hertfordshire, United Kingdom, Hatfield. http://sitem.herts.ac.uk/aeru/ppdb/en/.

[28] European Commission (2011). Commission Decision of 3 March 2011 concerning the non-inclusion of ethoxyquin in Annex I to Council Directive 91/414/EEC and amending Commission Decision 2008/941/EC. Off. J. Eur. Union.

[29] European Commission (2009). Commission Regulation (EC) No. 1107/2009 of the European Parliament and of the Council of 21 October 2009 concerning the placing of plant protection products on the market and repealing Council Directives 79/117/EEC and 91/414/EEC. Off. J. Eur. Union.

[30] Craig P.S., Dujardin B., Hart A., Hernandez-Jerez A.F., Hougaard Bennekou S., Kneuer C., Ossendorp B., Pedersen R., Wolterink G., Mohimont L. (2020). Cumulative dietary risk characterisation of pesticides that have chronic effects on the thyroid. EFSA J..

[31] European Commission (2002). Commission Regulation (EC) No. 2076/2002 of 20 November 2002 extending the time period referred to in Article 8(2) of Council Directive 91/414/EEC and concerning the non-inclusion of certain active substances in Annex I to that Directive and the withdrawal of authorisations for plant protection products containing these substances. Off. J. Eur. Union.

